# Supplementary material for: Schemas provide a scaffold for neocortical integration of new memories over time
Source: Nat Commun. 2022 Oct 2;13:5795. doi: 10.1038/s41467-022-33517-0 (PMC9527246; doi:10.1038/s41467-022-33517-0)
Supplement: Supplementary file 1 — Supplementary Information [file 41467_2022_33517_MOESM1_ESM.docx]

**Supplementary Information**

**Schemas provide a scaffold for neocortical integration of new memories over time**

Sam Audrain^1,2*^& Mary Pat McAndrews^1,2^

Division of Clinical and Computational Neuroscience, Krembil Research Institute, University Health Network, Toronto, ON, M5T 2S8, Canada;

2 Department of Psychology, University of Toronto, Toronto, ON, M5S 3G3, Canada

*Correspondence: [samantha.audrain@mail.utoronto.ca](mailto:samantha.audrain@mail.utoronto.ca), twitter: @samaudrain

**Supplementary Method 1: Proportion of ‘Don’t Know’ versus Incorrect responses.**

We calculated the proportion of trials for which participants explicitly responded that they didn’t know which context or scene had been presented with the object (‘don’t know’) versus chose the incorrect context or scene (‘incorrect’). We present a plot detailing the proportion of each type of response in **Supplementary Fig.1**.

**Supplementary Fig. 1. Proportion of forgotten trials where participants indicated “don’t know” versus responded incorrectly.** For context memory, % forgotten refers to the percent of total congruent or incongruent trials (as identified at encoding) where participants forgot which context the object had been paired with (don’t know = they indicated they did not know which context the object had been paired with; incorrect = they indicated the incorrect context was paired with the presented object). For scene memory, % forgotten refers to the percent of total congruent or incongruent trials (as identified at encoding) where participants remembered the correct context the object had been paired with, but did not remember the correct scene (don’t know = they indicated they did not know which scene the object had been paired with; incorrect = they indicated the incorrect scene was paired with the presented object). *N* = 23 participants across the short delay and *N* = 19 participants across the long delay. Errors bar reflect standard error of the mean adjusted for within-subject design. Source data are provided as a Source Data file.

**Supplementary Method 2: Proportional forgetting**

As there were more detailed congruent trials remembered across the short delay than detailed incongruent (t(57.5)=3.19, p=0.002), we considered the possibility that the increase in coarse congruent memory over time may be due to a larger number of detailed congruent memories available to decay into coarser memories. We therefore examined proportional forgetting across the long delay relative to the short delay, in order to control for different trial numbers across the short delay. We used the following formula to calculate proportion forgotten for each participant, as we have used in previous work ^1^:

Proportion forgotten = (forgetting across the short delay – forgetting across the long delay) / forgetting across the short delay

Positive proportional forgetting scores indicate greater forgetting across the long delay relative to the short delay, while negative scores indicate memory improvement across the long delay relative to the short delay. Scores around zero indicate no change in forgetting across the two delays. We fit a linear mixed model to proportion forgotten scores, with congruency (congruent/incongruent) and granularity (coarse/detailed) as predictors, with a random intercept for each participant and random slopes for counterbalancing groups. We note here that the model residuals were quite negatively skewed due to some extreme values in the coarse congruent condition. Specifically, three participants showed a very large increase in coarse congruent memory across the long delay (i.e. large negative proportion forgotten values). As data transformations did not normalize the model residuals, we omitted these participants’ scores for coarse congruent memory, and modelled the residual heteroskedasticity by adding weights using the ‘nlme’ package in R. **Supplementary Fig. 2** shows a plot of proportion forgotten for congruent and incongruent trails according to memory quality, excluding the 3 extreme values for the coarse congruent condition at the long delay.

Consistent with our analysis on percent correct behavioural data (Fig. 2 of the main manuscript), we found main effects of congruency (*F*(1,49) =10.93, *p* = 0.002), granularity (*F*(1,49) = 40.30, *p* < 0.0001), and a significant interaction between the two (*F*(1,49) = 4.23, *p* = 0.045). Post-hoc pairwise comparisons indicated greater forgetting of detailed memories than coarse for both congruent (coarse congruent: M = -1.04, SD = 0.91, detailed congruent: M = 0.33, SD = 0.48, *t*(49) = 5.29, *p* < 0.0001) and incongruent conditions (coarse incongruent: M = -0.23, SD = 0.60, detailed incongruent: M = 0.49, SD = 0.53, *t*(49) = 4.51, *p* < 0.0001). Participants generally forgot detailed memories (i.e. had positive forgetting scores), with greater forgetting in the detailed incongruent condition compared to the detailed congruent condition (fine incongruent: M = 0.49, SD = 0.53, fine congruent: M = 0.33, SD = 0.48, *t*(49) = 2.90, *p* = 0.006). In contrast, coarse memories generally increased over time (as indicated by the negative forgetting scores), and critically, there was a greater increase in coarse congruent than coarse incongruent memories (coarse congruent: M = -1.04, SD = 0.91, coarse incongruent: M = -0.23, SD = 0.60, *t*(49) = 2.60, *p* = 0.012). Thus this proportional forgetting analysis is in line with the percent correct analysis presented in the main manuscript. Specifically, while detailed memories are forgotten in both congruent and incongruent conditions, there is a greater increase in coarse memories over time in the congruent than incongruent condition. The observed increase in coarse congruent memory over time therefore cannot be accounted for by differences in number of trials retained across shorter delays.

**Supplementary Fig. 2.** **Proportion of coarse and detailed memories forgotten across the long delay relative to the short delay.** Positive values indicate greater forgetting over time, while negative values indicate an increase in memory over time. There was greater forgetting of detailed memories than coarse for both congruent (*t*(49) = 5.29, *p* < 0.0001, M difference = -1.43, CI[-1.99-(-0.90)]) and incongruent conditions (*t*(49) = 4.51, *p* < 0.0001, M difference = -0.78, CI[-1.13-(-0.43)]. Participants generally forgot detailed memories (i.e. had positive forgetting scores), with greater forgetting in the detailed incongruent condition compared to the detailed congruent condition (*t*(49) = 2.90, *p* = 0.006, M difference = -0.16, CI[-0.28-(-0.05)]. In contrast, coarse memories generally increased over time (as indicated by the negative forgetting scores), and critically, there was a greater increase in coarse congruent than coarse incongruent memories (*t*(49) = 2.60, *p* = 0.012, M difference = -0.83, CI[-1.47-(-0.19)]. *significant difference, according to uncorrected post-hoc two-tailed paired *t*-tests, descriptive of a significant interaction between congruency and granularity in the omnibus linear mixed effects model (*F*(1,49) = 4.23, *p* = 0.045, M difference = -0.66, CI[-1.31-(-0.02)]). *N* = 23 participants across the short delay and *N* = 19 participants across the long delay. Errors bar reflect standard error of the mean adjusted for within-subject design. Source data are provided as a Source Data file.

**Supplementary Method 3: Congruency bias analysis**

We considered the notion that an increase in congruency bias over time may explain the increase in coarse congruent memory observed over time, as well as change in pattern similarity in the mPFC for congruent versus incongruent trials. When participants are presented with a beach or kitchen object, they may be biased to choose the congruent context in the absence of veridical memory, thereby inflating coarse congruent memory scores. While a congruency bias would not be unexpected given past research ^2^, if congruency bias interacted with delay then that could change the interpretation of our results regarding change in quality of memory and pattern similarity over time. For example, it is possible that participants tend to be “lured” by the congruent context more over time as they forget, in which case an increase in pattern similarity for congruent trials could be due to increased guessing or an increase in false memory.

In order to assess congruency bias in our sample, we examined incorrect trials in the incongruent condition. Half of the incongruent trials consisted of context-related objects (e.g. beach objects paired with kitchens), and half were arbitrary objects paired with the contexts. We asked the question, were participants more likely to choose the incorrect but congruent context when it was available than to choose the incorrect context in the absence of congruency? This would be expected if congruency bias was influencing responding. We calculated congruency bias for each participant using the following equation:

Congruency bias = (Number of context-related object trials incorrect – number of arbitrary object trials incorrect)/total number of forgotten incongruent trials

We note here that the total number of forgotten incongruent trials includes all of the incorrect incongruent trials, as well as situations where they chose “don’t know” for the context question. Positive values indicate a congruency bias, such that participants were more likely to choose the incorrect context that was congruent with the presented beach or kitchen object, than they were to choose the incorrect context when presented with an arbitrary object (which was incongruent with both context options). Negative values indicate that participants were less likely to incorrectly choose the congruent context when presented with a beach or kitchen object, than they were to choose the incorrect context for an arbitrary object, possibly indicating better memory for incongruent context-related object trials than arbitrary object trials. Scores around zero indicate no bias one way or the other. **Supplementary Fig.3** displays a plot of congruency bias scores according to delay.

We ran one-sample *t*-tests at each delay to determine if congruency bias scores were reliably different from zero (i.e. no congruency bias), and found that there was indeed a positive congruency bias across both delays (short: M = 0.20, SD = 0.25, *t*(22) = 2.79, *p* = 0.011; long: M = 0.18, SD = 0.28, *t*(18) = 3.49, *p* = 0.003), indicating that participants were biased to choose the congruent context in response to context-related objects when it was available. We next subjected the congruency bias scores to a linear mixed effects model with delay as a predictor and a random intercept for each participant. There was no reliable difference in congruency bias across the short and long delay (*t*(21) = 0.16, *p* = 0.88). In other words, participants showed a congruency bias across both delays such that they were more likely to choose the congruent context when presented with a beach or kitchen object. However, this bias did not proportionally increase over time, and so is unlikely to explain the change in memory quality and pattern similarity we observe over time in the congruent condition.


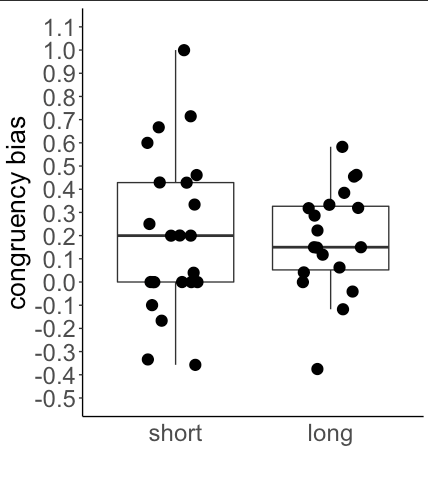


**Supplementary Fig. 3. Congruency bias across the short and long delay.** Positive values indicate a congruency bias associated with more incorrect trials, negative values indicate congruency bias associated with fewer incorrect trials, scores around zero indicate no congruency bias. Center line reflects the median, upper and lower box bounds reflect 75^th^ and 25^th^ percentiles respectively, upper and lower whiskers reflect maximum and minimum values excluding outliers. Black dots represent scores from individual participants. *N* = 23 participants across the short delay and *N* = 19 participants across the long delay. Source data are provided as a Source Data file.

**Supplementary Method 4: Comparison of connectivity-memory correlations**

We sought to test the specificity of the relationship between post-encoding anterior hippocampus-mPFC connectivity and coarse congruent memory. To do so, we directly tested the difference between our a priori hypothesized correlation and correlations between connectivity and the other memory conditions using William’s test for dependent correlations.

We found that the correlation between post-encoding connectivity and coarse congruent memory was not reliably different from that for coarse incongruent memories (*t* = 0.92, *p* = 0.37), but was marginally different from that for detailed congruent memories (*t* = 2.03, *p* = 0.06), and was reliably different than that for detailed incongruent memories (*t* = 3.13, *p* = 0.01). Thus, while there was no reliable relationship between post-encoding connectivity and coarse incongruent memory (*r* = 0.19, as described in the main manuscript), the slope of this correlation was not reliably different from that for coarse congruent memories, leaving open the possibility that the relationship between post-encoding connectivity and coarse congruent memory is not specific to the congruent condition, but instead is associated with coarse memory in general over time. However, the absence of a relationship between coarse incongruent memory and post-encoding connectivity obscures the validity of this interpretation. The slopes for all four of these correlations overlaid together can be viewed in **Supplementary Fig. 4**.


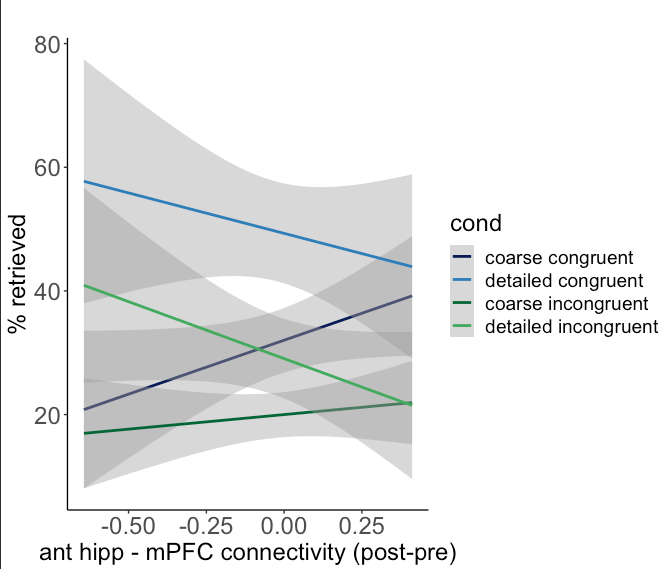


**Supplementary Fig. 4. Correlations between change in post-encoding anterior hippocampus-mPFC connectivity and % retrieved 72-hours later.** Memories were considered coarse if participants retrieved the correct context an object had been paired with but not the specific scene and were considered detailed if they retrieved the specific scene. Colored lines reflect lines of best fit for each correlation. Grey ribbon represents 95% confidence interval. Source data are provided as a Source Data file.

**Supplementary Method 5: Pattern similarity in the hippocampus over time during retrieval of object-context pairs, regardless of memory granularity.**

We ran the same pattern similarity analysis in the hippocampus as conducted for the mPFC (Fig. 4 of the main manuscript). These results therefore reflect pattern similarity of coarse features of memory in the hippocampus, according to congruency (**Supplementary Fig.5**). A linear mixed model predicting pattern similarity in the posterior hippocampus with context (within/across), congruency (congruent/incongruent) and delay (short/long) indicated a significant congruency by delay interaction (*F*(1,28168) = 8.29, *p* = 0.004). There was also a significant congruency by delay interaction in the anterior hippocampus (*F*(1,28168) = 25.77, *p* < 0.0001).

**Supplementary Fig. 5. Pattern similarity in the hippocampus over time during retrieval of congruent and incongruent object-context pairs.** Patterns for trials where participants successfully retrieved the context associated with the presented object (regardless of memory quality) were extracted from the hippocampus and correlated within and across context, separately for congruent and incongruent trials. Data reflect estimated marginal means from linear mixed effects models predicting pairwise Fisher transformed correlations from congruency, context, and delay variables, in *N* = 23 participants across the short delay and *N* = 19 participants across the long delay. Errors bar reflect standard error of the mean adjusted for within-subject design. Source data are provided as a Source Data file. Within=within-context correlations; across=across-context correlations.

**Supplementary Method 6:** **Object Similarity Analysis**

We considered the possibility that the observed increase in pattern similarity for congruent object-context pairs in the mPFC (Fig. 4 of the main manuscript) could be due to the fact that objects in the congruent condition are more semantically similar to each other than they are in the incongruent condition. In other words, perhaps the increase in pattern similarity over time was not driven by congruency between the object and context, but rather reflects congruency between objects within kitchen and beach categories. We therefore isolated the effect of object congruency from the effect of context congruency by examining pattern similarity for correct incongruent trials. We computed two sets of correlations: 1) pattern similarity between **arbitrary objects** that shared the same incongruent context (objects that were not related to kitchens or beaches), and 2) pattern similarity between **context-related objects** that shared the same incongruent context (objects related to kitchens were correlated with each other, and objects related to beaches were correlated with each other; **Supplementary Fig. 6a**). Notably, all of these object-context pairs were incongruent, so any observed difference in pattern similarity between the arbitrary and context-related objects should reflect congruency (or lack thereof) between objects, independent of congruency with the background.

We submitted these correlations to a linear mixed model with object type (arbitrary/context-related) and delay (short/long) as independent variables, with a random intercept for each participant and random slope for each counterbalancing condition. We found a main effect of delay (*F*(1,5050) = 21.91, *p* < 0.0001), and no main effect of object type (*F*(1,5050) = 1.49, *p* = 0.22). There was also a significant interaction between object type and delay (*F*(1,5050) = 18.58, *p* < 0.0001). Pairwise *t*-tests indicated that at the short delay, patterns for context-related objects were more similar to each other than were patterns for arbitrary objects (*t*(5050) = 3.22, *p* = 0.001), while at the long delay, patterns for arbitrary objects were more similar to each other than were context-related objects (*t*(5050) = 3.17, *p* = 0.002). Interestingly, while pattern similarity for context-related objects did not change over time (*t*(5050) = 0.88, *p* = 0.38), patterns for arbitrary objects became more similar to each other (*t*(5050) = 6.30, *p* < 0.0001; **Supplementary Fig. 6b**). Thus, while patterns for objects from the same schema were more similar to each other than were patterns for arbitrary objects early on in the mPFC, such representations were not further integrated over time in the absence of a congruent background. In fact, it was the arbitrary objects that came to be represented more similarly over time. These results support the interpretation that congruent object-context pairs are integrated in the mPFC according to shared congruent context, rather than semantic similarity between objects.

**Supplementary Fig. 6. Representational pattern similarity based on object congruency.** (a) Patterns for incongruent trials where participants successfully retrieved the context associated with the presented object (regardless of memory quality) were extracted from the mPFC and correlated according to object congruency. Specifically, patterns were correlated between arbitrary objects that shared the same incongruent context (objects that were not related to kitchens or beaches), and between context-related objects that shared the same incongruent context (objects related to kitchens were correlated with each other, and objects related to beaches were correlated with each other). (b) Resulting pattern similarity in the mPFC over time, according to object congruency (arbitrary/context-related). Pairwise *t*-tests indicated that at the short delay, patterns for context-related objects were more similar to each other than were patterns for arbitrary objects (*t*(5050) = 3.22, *p* = 0.001, M difference = -0.012, CI[-0.019-(-0.005)]), while at the long delay, patterns for arbitrary objects were more similar to each other than were context-related objects (*t*(5050) = 3.17, *p* = 0.002, M difference = 0.023, CI[0.009-0.038]). Patterns for arbitrary objects became more similar to each other over time (*t*(5050) = 6.30, *p* < 0.0001, M difference = 0.030, CI[0.020-0.039]). Data reflect estimated marginal means from a linear mixed effects model predicting pairwise Fisher transformed correlations from object type and delay variables, in *N* = 23 participants across the short delay and *N* = 19 participants across the long delay. Errors bar reflect standard error of the mean adjusted for within-subject design. *significant difference, according to uncorrected post-hoc two-tailed paired *t*-tests, describing a significant interaction between object type and delay from an omnibus linear mixed effects model (*F*(1,5050) = 18.58, *p* < 0.0001, M difference = 0.036, CI[0.019-0.052]). Scene and object images presented here are placeholders used for illustrative purposes. Objects were retrieved from the bank of standardized stimuli (BOSS) database (Copyright (C) 2009, 2010 Mathieu Brodeur)^3^. Beach photos by Rowan Heuvel and Pedro Monteiro on Unsplash: <https://unsplash.com/license>. Source data are provided as a Source Data file. r=Pearson’s correlation.

**Supplementary Method 7: Integration of arbitrary objects only**

One reason why we may not have observed a difference between within and across context correlations for the incongruent condition in the mPFC over time (i.e. context-specific integration) may be due to some residual semantic overlap between context-related objects that had been paired with incongruent scenes (e.g. oven mitt – beach) and the opposing context with which it was correlated in the across-context correlations (objects paired with kitchens). In other words, it could be the case that semantic overlap between context-related objects and opposing contexts with which they are related could inflate correlations in the across-context condition, thereby reducing a difference between within and across-context correlations. To test this, we excluded context-related objects (i.e. objects related to kitchens or beaches) in the incongruent condition from the analysis. With the remaining arbitrary objects (objects unrelated to kitchens or beaches) we re-computed two sets of correlations: 1) pattern similarity between arbitrary objects that shared the same incongruent contexts (**within-context similarity**) and 2) pattern similarity between arbitrary objects that had been paired with opposing contexts (**across-context similarity; Supplementary Fig. 7a**). If a within versus across context difference in pattern similarity should emerge across the long delay for these incongruent arbitrary object-context pairs, it would signal that semantic overlap between objects and opposing contexts may have masked a true effect of integration of incongruent pairs in the mPFC over the 3 days.

We ran a linear mixed effects model predicting pattern similarity as a function of context (within/across) and delay (short/long), with a random intercept modelled for each participant and random slopes for counterbalancing condition. We observed a main effect of delay (*F*(1,6228) = 47.95, *p*< 0.0001) but no effect of context (*F*(1,6228) = 0.19, *p*= 0.67), and no delay by context interaction (*F*(1,6228) = 2.08, *p* = 0.15). Thus, we did not observe evidence that residual semantic overlap in the across-context condition was driving the lack of a difference between within and across context correlations in the mPFC for incongruent information.

**Supplementary Fig. 7.** **Representational similarity analysis in the mPFC during retrieval of incongruent arbitrary object-context pairs (excluding context-related objects)**

**(a)** Schematic example of our analysis approach. Patterns for trials where participants successfully retrieved the context associated with the presented arbitrary object (regardless of memory quality) were extracted from the mPFC and correlated within and across context for incongruent trials. Context-related objects (i.e. objects related to kitchens or beaches) were excluded from this analysis. Background scenes were not presented during retrieval, but were retrieved from memory **(b)** Resulting pattern similarity in the mPFC over time, according to context (within/across). Data reflect estimated marginal means from a linear mixed effects model predicting pairwise Fisher transformed correlations from context and delay variables, in *N* = 23 participants across the short delay and *N* = 19 participants across the long delay. Errors bar reflect standard error of the mean adjusted for within-subject design. Scene and object images presented here are placeholders used for illustrative purposes. Objects were retrieved from the bank of standardized stimuli (BOSS) database (Copyright (C) 2009, 2010 Mathieu Brodeur) ^3^. Beach photos by Rowan Heuvel and Pedro Monteiro, kitchen photos by Sidekix Media and Zac Gudakov, all on Unsplash: <https://unsplash.com/license>. Source data are provided as a Source Data file. r=Pearson’s correlation.

**Supplementary Method 8: Pattern similarity within and across contexts in the mPFC restricted to detailed memory trials.**

Patterns for trials where participants successfully retrieved the correct scene (detailed memory trials) associated with the presented object were extracted from the mPFC and correlated according to if the objects had been paired with the same context (within-context correlations) or opposing context (across-context correlations), within congruency (Fig. 4 of the main manuscript, but here restricted to detailed trials).

A linear mixed effects model predicting pattern similarity for within-context correlations as a function of congruency (congruent/incongruent) and delay (short/long) revealed a significant interaction between congruency and delay (*F*(1,7952) = 4.25, *p* = 0.039), suggesting that even detailed congruent trials become more similar to each other than incongruent in the mPFC over time (**Supplementary Fig. 8**; “congruent within” and “incongruent within” bars). We further assessed if integration was specific to the context with objects were learned by running linear mixed effects models predicting pattern similarity as a function of context (within/across) and delay (short/long) separately for congruent and incongruent trials. We found there was no longer a main effect of context as observed in the main manuscript (*F*(1,9910) = 1.01, *p* = 0.32), but there was a marginal interaction between context and delay (*F*(1,9910) = 3.48, *p* = 0.062) that appears to be driven by greater within than across context integration at the long delay (“congruent within” and “congruent across” bars **in Supplementary Fig. 8**). Thus, there may be some context-specific integration happening for detailed congruent memories. There was no effect of context for incongruent trials (*F*(1,6262) = 0.46, *p* = 0.50), and no context by delay interaction (*F*(1,6262) = 0.86, *p* = 0.35; “incongruent within” and “incongruent across” bars in **Supplementary Fig. 8**).

**Supplementary Fig. 8.** **Pattern similarity in the mPFC over time during retrieval of detailed congruent and incongruent object-scene pairs.** Data reflect estimated marginal means from linear mixed effects models predicting pairwise Fisher transformed correlations from congruency, context, and delay variables, in *N* = 23 participants across the short delay and *N* = 19 participants across the long delay. Errors bar reflect standard error of the mean adjusted for within-subject design. Source data are provided as a Source Data file. Within=within-context correlations, across=across-context correlations.

**Supplementary Method 9: Relationship between connectivity and integration.**

In order to determine if post-encoding connectivity between the anterior hippocampus and mPFC was related to representational integration in the mPFC for congruent trails across the long delay, we averaged pattern similarity correlations for congruent trials at the long delay separately for within-context and across-context conditions, within each participant. We then subtracted the average across-context correlation from the average within-context correlation for each participant, and correlated the resulting measure of congruent within vs across context pattern similarity with post-pre encoding connectivity between the anterior hippocampus and mPFC. We did not observe a relationship between post-encoding anterior hippocampus to mPFC connectivity and subsequent integration of congruent trials in the mPFC within versus across context (*r* = -0.21, *t*(15) = 0.85, *p* = 0.41).

**Supplementary Method 10: Pattern similarity of forgotten trials**

If observed differences in pattern similarity between conditions is truly driven by memory, we should not observe the same pattern of results for forgotten trials. To this end, we re-ran the same analyses reported in the main manuscript using forgotten trials. Briefly, we extracted the pattern of voxels in each ROI (mPFC/posterior hippocampus/anterior hippocampus) as participants were viewing objects and failing to successfully retrieve the associated context. For the mPFC analysis, we correlated the extracted voxel patterns with all other forgotten trails that

that had shared the same context at encoding (forgotten within context correlations), and with those forgotten congruent trials of opposing contexts at encoding (forgotten across context correlations). For incongruent pairs, we computed the same set of correlations except all forgotten object-context pairs had been incongruent at encoding in keeping with the original mPFC analyses in the main manuscript. In the hippocampal ROIs, we correlated the forgotten trials based on if the object had been paired with the same scene, similar scene, or other context at encoding, separately for congruent and incongruent trials. To make these analyses as comparable as possible to the main manuscript, we re-ran the exact same statistical models as were run on remembered trials.

In the mPFC, we ran a linear mixed effects model predicting within-context pattern similarity of forgotten trials in the mPFC as a function of congruency and delay, with random intercepts for each participant and random slopes for each counterbalanced group. We found no main effects of congruency (*F*(1,1435) = 2.95, *p* = 0.086) or delay (*F*(1, 1435) = 0.37, *p* = 0.54) and no interaction between the two (*F*(1,1435) = 0.73, *p* = 0.39). Unlike for remembered trials, patterns during forgotten trials that had shared contexts at encoding did not become more similar to each other in the mPFC over time, regardless of congruency. We additionally computed a linear mixed model predicting pattern similarity as a function of context (same context/across context) and delay (short/long), separately for congruent and incongruent pairs, as done in the main manuscript for remembered trials. Random intercepts were included for each participant and random slopes for each counterbalanced group. We found that for congruent pairs there was a main effect of context due to greater similarity of patterns within than across context (*F*(1,340) = 4.50, *p* = 0.035), as we found for remembered trials. There was no effect of delay (*F*(1,340) = 0.06, *p* = 0.81) and no context by delay interaction (*F*(1,340) = 1.36, *p* = 0.25). We note here that while it may appear that context was represented in the mPFC for forgotten congruent trials, this finding was driven primarily by a difference in pattern similarity between within and across context correlations for forgotten trials across the short delay (*t*(340) = 1.83, *p* = 0.068), where participants only forgot 4.48 trials on average (SD = 4.44), and was not driven by a difference across the long delay (within vs across context at long delay: *t*(340) = 1.08, *p* = 0.28). This finding should therefore not be taken as evidence of context specificity for forgotten congruent trials, due to too few forgotten congruent trials with which to measure this effect across the short delay. For incongruent trials, there was no effect of context (*F*(1,2497) = 2.43, *p* = 0.12), delay (*F*(1,2497) = 0.04, *p* = 0.84), and no context by delay interaction (*F*(1,2497) = 0.53, *p* = 0.47).

For each hippocampal ROI, we submitted forgotten correlations to a scene (same/similar/other scene) x congruency (congruent/incongruent) x delay (short/long) linear mixed model, with a random intercept for each participant and random slopes for the counterbalanced groups. There were no significant main effects or interactions in either the posterior or anterior hippocampi (**Supplementary Table 1**), the absence of which suggests that the pattern similarity findings observed in the main manuscript do in fact reflect memory.

Finally, we tested for differences in pattern similarity between remembered and forgotten trials directly, using linear mixed effects models to predict pattern similarity in each ROI as a function of memory status (remembered/forgotten), congruency (congruent/incongruent), and condition (within/across context correlations for the mPFC, same/similar/other detailed scene correlations for the hippocampal ROIs). We restricted the analysis to the long delay where the effects of interest in the main manuscript reside given the small number of forgotten trials across the short delay. However, we plot the data across both delays for visual comparison (**Supplementary Fig. 9**). Results of these models can be observed in Supplementary Table 2. Importantly, a main effect of memory status (remembered vs forgotten) is significant for all three ROIs at the long delay, and therefore remembered and forgotten trials have different pattern similarity on average.

**Supplementary Table 1.** Results of hippocampal pattern similarity models on forgotten trials over time. Linear mixed effects models predicted pairwise Fisher transformed correlations with congruency (congruent/incongruent), delay (short/long), and scene variables (same/similar/other scene) as predictors. A random intercept for each participant and a random slope for counterbalancing conditions was included. P values are uncorrected for multiple comparisons. DFn=degrees of freedom for the numerator; DFd=degrees of freedom for the denominator.

|  | | | | |
| --- | --- | --- | --- | --- |
| **Effects** | **DFn** | **DFd** | **F** | **P** |
| **Posterior Hippocampus** |  |  |  |  |
| scene | 2 | 4759 | 2.06 | 0.13 |
| congruency | 1 | 4759 | 0.08 | 0.78 |
| delay | 1 | 4759 | 0.02 | 0.90 |
| scene x congruency | 2 | 4759 | 0.39 | 0.67 |
| scene x delay | 2 | 4759 | 2.2 | 0.11 |
| congruency x delay | 1 | 4759 | 0.002 | 0.97 |
| scene x congruency x delay | 2 | 4759 | 2.54 | 0.08 |
| **Anterior Hippocampus** |  |  |  |  |
| scene | 2 | 4759 | 1.21 | 0.30 |
| congruency | 1 | 4759 | 0.04 | 0.84 |
| delay | 1 | 4759 | 0.43 | 0.51 |
| scene x congruency | 2 | 4759 | 2.18 | 0.11 |
| scene x delay | 2 | 4759 | 1.05 | 0.35 |
| congruency x delay | 1 | 4759 | 0.02 | 0.88 |
| scene x congruency x delay | 2 | 4759 | 0.20 | 0.82 |
|  |  |  |  |  |

**Supplementary Fig. 9.** **Pattern similarity for remembered compared to forgotten trials.** (a) Resulting pattern similarity for remembered and forgotten trials in the mPFC over time, according to congruency (congruent/incongruent) and context (within/across). (b) Resulting pattern similarity in the posterior and (c) anterior hippocampus for remembered and forgotten trials over time, according to scene overlap. Data reflect estimated marginal means from linear mixed effects models predicting pairwise Fisher transformed correlations from congruency, memory status, and context or scene variables, in *N* = 23 participants across the short delay and *N* = 19 participants across the long delay. Error bars reflect standard error of the mean adjusted for within-subject design. Note that there were few trials forgotten in the congruent condition across the short delay (M=4.48 trials forgotten, SD=4.44), likely contributing to large variability in this condition. Source data are provided as a Source Data file.

**Supplementary Table 2. Results of pattern similarity models on remembered and forgotten trials across the long delay.** Linear mixed effects models predicted pairwise Fisher transformed correlations with memory status (remembered/forgotten), congruency (congruent/incongruent), and context (within/across, for mPFC) or scene variables (same/similar/other scene, for hippocampal ROIs) as predictors for data acquired across the long delay (*N* = 19). A random intercept for each participant and a random slope for counterbalancing conditions was included. P values are uncorrected for multiple comparisons. DFn=degrees of freedom for the numerator; DFd=degrees of freedom for the denominator.

| **Effects** | **DFn** | **DFd** | **F value** | **P value** |
| --- | --- | --- | --- | --- |
| **mPFC** | | | | |
| Memory status | 1 | 11375 | 102.09 | <0.000001 |
| Congruency | 1 | 11371 | 15.20 | 0.000097 |
| Context | 1 | 11365 | 1.23 | 0.27 |
| Memory status x congruency | 1 | 11379 | 7.29 | 0.0069 |
| Memory status x context | 1 | 11365 | 0.29 | 0.59 |
| Congruency x context | 1 | 11365 | 4.82 | 0.028 |
| Memory x congruency x context | 1 | 11365 | 1.09 | 0.30 |
| **Posterior hippocampus** | | | | |
| Memory status | 1 | 6232.6 | 54.65 | <0.000001 |
| Congruency | 1 | 6677.9 | 1.54 | 0.21 |
| Scene | 2 | 6707.2 | 2.45 | 0.086 |
| Memory status x congruency | 1 | 6236.5 | 0.034 | 0.85 |
| Memory status x scene | 2 | 6707 | 1.35 | 0.26 |
| Congruency x scene | 2 | 6702.3 | 0.035 | 0.97 |
| Memory x congruency x scene | 2 | 6701.4 | 1.65 | 0.19 |
| **Anterior hippocampus** | | | | |
| Memory status | 1 | 6468.1 | 10.38 | 0.001 |
| Congruency | 1 | 6701.8 | 3.34 | 0.068 |
| Scene | 2 | 6706.2 | 0.88 | 0.41 |
| Memory status x congruency | 1 | 6465.9 | 0.31 | 0.58 |
| Memory status x scene | 2 | 6705.9 | 2.89 | 0.056 |
| Congruency x scene | 2 | 6701.1 | 0.81 | 0.45 |
| Memory x congruency x scene | 2 | 6700.4 | 1.13 | 0.32 |

**Supplementary Method 11: Pattern similarity broken down by scene category.**

**Supplementary Fig. 10.** **Pattern similarity for kitchen and beach categories**. Pattern similarity was recomputed in the a) mPFC, b) anterior hippocampus, and c) posterior hippocampus within kitchen and beach categories. Within context, same scene and similar scene correlations were restricted to beach or kitchen trials. Across context and other context correlations reflect correlations between kitchens and beaches (i.e. the same correlations as in the main manuscript for these conditions). Data reflect estimated marginal means from linear mixed effects models predicting pairwise Fisher transformed correlations from congruency, context, and delay in the mPFC, and from scene and delay in the hippocampal regions, in *N* = 23 participants across the short delay and *N* = 19 participants across the long delay. Error bars reflect standard error of the mean adjusted for within-subject design. Source data are provided as a Source Data file.

**Supplementary Method 12:** **Pattern similarity in the hippocampus and mPFC over time during retrieval of detailed congruent and incongruent object-scene pairs.**

We present the data from Fig. 5 of the main manuscript, expanded to show pattern similarity according to congruency (**Supplementary Fig.11**). We additionally include a plot of the same contrasts in the mPFC for comparison.

**Supplementary Fig. 11.** **Pattern similarity in the hippocampus and mPFC over time during retrieval of detailed congruent and incongruent object-scene pairs.** Patterns for trials where participants successfully retrieved both the context and scene associated with the presented object (detailed memories) were extracted from the hippocampus and mPFC and correlated according to if the objects had been paired with the same scene, similar scenes, or were paired with opposing/other contexts, within congruency. Data reflect estimated marginal means from linear mixed effects models predicting pairwise Fisher transformed correlations from congruency, scene, and delay variables, in *N* = 23 participants across the short delay and *N* = 19 participants across the long delay. Errors bar reflect standard error of the mean adjusted for within-subject design. Source data are provided as a Source Data file. Same = same scene correlations; sim = similar scene correlations; other = other/opposing scene correlations.

**Supplementary Method 13: Pattern similarity in the posterior hippocampus over time collapsed across detailed and coarse memory trials.**

Patterns for trials where participants successfully retrieved the correct context, regardless of if they retrieved the specific scene associated with the presented object, were extracted from the posterior hippocampus and correlated according to if the objects had been paired with the same scene, similar scenes, or were paired with opposing/other scene contexts, within congruency. If representational scene specificity in the posterior hippocampus is weaker or absent for coarse trials, the pattern observed in Fig. 5 of the main manuscript (of greater pattern similarity for object-same scene pairs than object-similar or -other scene pairs for detailed trials) should be attenuated when coarse memory trials are included in the analysis. We note here that there were too few coarse memory trials in each condition (short delay: related M = 7.26, SD = 4.43; unrelated M = 6.47, SD = 4.23; long delay: related M = 12.79, SD = 4.43; unrelated M = 7.47, SD = 2.91) to conduct this analysis on coarse trials exclusively.

A linear mixed effects model predicting pattern similarity as a function of scene (same/similar/other), congruency (congruent/incongruent) and delay(short/long) with random intercepts for each participant revealed a main effects of delay (*F*(1,28494) = 22.53, *p* < 0.0001) and a congruency x delay interaction (*F*(1,28736) = 8.59, *p* = 0.003). Unlike results restricted to detailed memory trials only, there was no effect of scene (*F*(2,28717) = 2.27, *p* = 0.10), and no scene by delay interaction (*F*(2,28717) = 0.27, *p* = 0.77). Thus, there was no representational scene specificity in the posterior hippocampus when coarse trials were included in the analysis (**Supplementary Fig.12**).

**Supplementary Fig. 12.** **Pattern similarity in the posterior hippocampus over time during retrieval of congruent and incongruent object-scene pairs collapsed across coarse and detailed memory trials.** Data reflect estimated marginal means from a linear mixed effects model predicting pairwise Fisher transformed correlations from congruency, scene, and delay variables, in *N* = 23 participants across the short delay and *N* = 19 participants across the long delay. Errors bar reflect standard error of the mean adjusted for within-subject design. Source data are provided as a Source Data file. Same = same scene correlations; sim = similar scene correlations; other = other/opposing scene correlations.

**Supplementary Method 14: Pattern similarity in the left hippocampus**

We focus on the right hippocampus in the main manuscript due to the well-documented involvement of the right hippocampus in retrieving visual memory. Here, we present pattern similarity results in the left hippocampus for the interested reader.

**Supplementary Fig. 13. Representational similarity analysis of object-scene pairs retrieved with detail in the left hippocampus.** Pattern similarity in the (a) left posterior and (b) left anterior hippocampus over time, according to scene overlap. Data reflect estimated marginal means from linear mixed effects models predicting pairwise Fisher transformed correlations from congruency, scene, and delay variables, in *N* = 23 participants across the short delay and *N* = 19 participants across the long delay. Errors bar reflect standard error of the mean adjusted for within-subject design. Source data are provided as a Source Data file. Same = correlations between objects that ha been paired with the same scene during encoding, sim = correlations between objects paired with similar scenes at encoding, other = correlations between object from opposing scene contexts at encoding.

**Supplemental Method 15: Power Analysis**

This study was powered on Tompary and Davachi’s (2017)^4^ finding of increased pattern similarity in the mPFC over time for arbitrary object-scene pairs that share the same/overlapping scene, versus were paired with different/non-overlapping scenes. Specifically, we powered the study based on the interaction the authors described between time and stimulus overlap on pattern similarity in the mPFC (*F*(1,18) = 7.33, *p* = 0.01), as the role of the mPFC in integrating memories was the primary aim of our study. As Tompary and Davachi (2017) did not report effect sizes for this analysis, we calculated partial eta-squared using their *F* statistic and degrees of freedom using the following formula:

Partial eta-squared = (*F* * DF1) / ((*F* * DF1) + DF2)

We calculated that the partial eta-squared for the effect of interest was 0.29, which is a large effect size for the interaction between time and stimulus overlap. As there is no way to convert partial eta squared to Cohen’s *f* required for G*Power analysis, and no way to calculate Cohen’s *f* based on the information provided in the Tompary and Davachi (2017) paper, we chose to approximate an effect size for the power analysis. Given the issue of publication bias and true effect sizes tending to be smaller than the original published findings, we used a smaller, medium effect size approximate for a Cohen’s *f*, of 0.25. In G*power, we used the Cohen’s f (0.25) to estimate a repeated measures ANOVA within factors, with 1 group, and 4 measurements (delay(2) x congruency(2)), with 0.5 correlation among repeated measures. This analysis indicated that 23.13 participants would achieve 80% power.

**Supplementary Method 16:** **Post-encoding anterior hippocampus-mPFC** **connectivity correlation with coarse congruent memory plotted according to counterbalancing group**

We identified individuals in each counterbalanced group to visually assess if the order in which participants were tested across short and long delays differently contributed to the correlation. Participants in Group A underwent testing across the short delay followed by the long delay, and participants in Group B underwent testing across the long delay followed by the short delay. Only 4 participants in Group A were included in this correlation analysis after data exclusions outlined in the Methods section of the main manuscript, and their datapoints are not noticeably different from that of participants in Group B (**Supplementary Fig.14**).


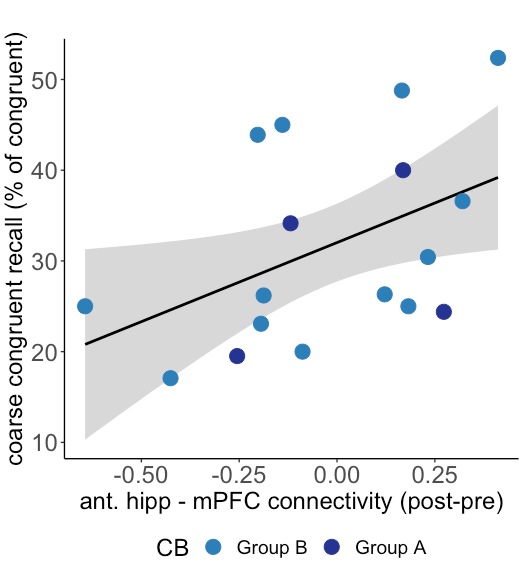


**Supplementary Fig. 14. Connectivity according to counterbalancing group.** Group A underwent testing across the short delay followed by the long delay. Group B underwent testing across the long delay followed by the short delay. The dots reflect individual participants, and the black line reflects the line of best fit for the correlation. The grey ribbon represents 95% confidence interval for a one-tailed test. Source data are provided as a Source Data file.

**Supplemental Method 17: Univariate Control Analysis**

To exclude the possibility that differences in univariate activation across congruency and delay were driving pattern similarity results ^5^, we extracted the univariate activation averaged across ROI (mPFC/anterior hippocampus/posterior hippocampus) for each trial, and modelled univariate estimates as a function of congruency of the trials (congruent/incongruent) and delay (short/long) separately for each ROI using linear mixed models with random intercepts for each participant and random slopes for counterbalancing condition. To be consistent with the analyses in the main manuscript, we included the beta estimates for all correctly retrieved trials regardless of quality of memory in our mPFC model, and all correctly retrieved detailed memory trials in our anterior and posterior hippocampus models. We note here that it is not straightforward to simply regress trial-wise univariate activity out of the RSA models in this case, because we modelled the correlations between trials rather than average pattern similarity per trial (i.e. each correlation is the product of two trials, each with corresponding univariate activity). However, should any effects on univariate activation mirror the effects observed in our RSA analyses it might indicate that such effects were driven by differences in univariate activation rather than pattern similarity.

In the mPFC there was no effect of delay (*F*(1,2381.9) = 0.45, *p* = 0.50), congruency (*F*(1,2445.9) = 0.81, *p* = 0.37), or an interaction between the two (*F*(1,2442.9) = 2.05, *p* = 0.15) on trial-wise activation. Likewise, univariate activation for trials retrieved with detail did not reliably vary as a function of congruency or delay in the anterior (congruency: *F*(1,1747.9) = 0.66, *p* = 0.42; delay: *F*(1,1707.3) = 0.03, *p* = 0.87; interaction: *F*(1,1749.1) = 0.21, *p* = 0.64) or posterior hippocampus (congruency: *F*(1,1745.5) = 2.37, *p* = 0.12; delay: *F*(1,1702.2) = 0.03, *p* = 0.85; interaction: *F*(1,1747.7) = 0.45, *p* = 0.50). It is therefore unlikely that any of our results are driven by differences in univariate activation based on congruency of the object-scene pairs or delay.

**Supplementary Method 18. Visualizing pairwise correlations.**

We overlaid the individual pairwise correlations contributing to the bar plots in Figures 4 and 5 of the main manuscript to assess for outliers that could be driving observed effects. Aberrant datapoints do not appear to be driving effects of interest.

**Supplementary Fig. 15. Visualizing pairwise correlations of interest.** Pairwise correlations contributing to the bar plots in Figures 4 and 5 of the main manuscript were plotted and inspected for outliers that could be driving observed effects. Data reflect Fisher transformed pairwise correlations between trials from *N* = 23 participants across the short delay and *N* = 19 participants across the long delay. Source data are provided as a Source Data file.

**Supplementary References**

1. Audrain, S. & McAndrews, M. P. Cognitive and functional correlates of accelerated long-term forgetting in temporal lobe epilepsy. *Cortex* **110**, 101–114 (2019).

2. Pardilla-Delgado, E. & Payne, J. D. The Deese-Roediger-McDermott (DRM) Task: A Simple Cognitive Paradigm to Investigate False Memories in the Laboratory. *J. Vis. Exp.* **2017**, 1–10 (2017).

3. Brodeur, M. B., Dionne-Dostie, E., Montreuil, T. & Lepage, M. The bank of standardized stimuli (BOSS), a new set of 480 normative photos of objects to be used as visual stimuli in cognitive research. *PLoS One* **5**, e10773 (2010).

4. Tompary, A. & Davachi, L. Consolidation promotes the emergence of representational overlap in the hippocampus and medial prefrontal cortex. *Neuron* **96**, 228-241.e5 (2017).

5. Dimsdale-Zucker, H. R. & Ranganath, C. Representational Similarity Analyses: A Practical Guide for Functional MRI Applications. *Handb. Behav. Neurosci.* **28**, 509–525 (2018).
