# Supplementary material for: Schemas provide a scaffold for neocortical integration of new memories over time
Source: Nat Commun. 2022 Oct 2;13:5795. doi: 10.1038/s41467-022-33517-0 (PMC9527246; doi:10.1038/s41467-022-33517-0)
Supplement: Supplementary file 3 — Reporting Summary [file 41467_2022_33517_MOESM3_ESM.pdf]

nature portfolio

Corresponding author(s): Sam Audrain

Last updated by author(s): Aug 10, 2022

Reporting Summary

Nature Portfolio wishes to improve the reproducibility of the work that we publish. This form provides structure for consistency and transparency in reporting. For further information on Nature Portfolio policies, see our Editorial Policies and the Editorial Policy Checklist.

Statistics

For all statistical analyses, confirm that the following items are present in the figure legend, table legend, main text, or Methods section.

n/a	Confirmed	
☐	☒	The exact sample size (*n*) for each experimental group/condition, given as a discrete number and unit of measurement
☐	☒	A statement on whether measurements were taken from distinct samples or whether the same sample was measured repeatedly
☐	☒	The statistical test(s) used AND whether they are one- or two-sided<br>*Only common tests should be described solely by name; describe more complex techniques in the Methods section.*
☐	☒	A description of all covariates tested
☐	☒	A description of any assumptions or corrections, such as tests of normality and adjustment for multiple comparisons
☐	☒	A full description of the statistical parameters including central tendency (e.g. means) or other basic estimates (e.g. regression coefficient) AND variation (e.g. standard deviation) or associated estimates of uncertainty (e.g. confidence intervals)
☐	☒	For null hypothesis testing, the test statistic (e.g. *F*, *t*, *r*) with confidence intervals, effect sizes, degrees of freedom and *P* value noted<br>*Give P values as exact values whenever suitable.*
☒	☐	For Bayesian analysis, information on the choice of priors and Markov chain Monte Carlo settings
☐	☒	For hierarchical and complex designs, identification of the appropriate level for tests and full reporting of outcomes
☐	☒	Estimates of effect sizes (e.g. Cohen's *d*, Pearson's *r*), indicating how they were calculated

Our web collection on statistics for biologists contains articles on many of the points above.

Software and code

Policy information about availability of computer code

Data collection

Custom behavioural experimental code, administration, and collection was performed using Inquisit 5 by Millisecond: https://www.millisecond.com/

All scanning was performed using a Siemens Prisma 3T full-body MRI scanner, using custom sequences.

Data analysis

The resting state scans were preprocessed and modelled using CONN version 18b (https://web.conn-toolbox.org/), which utilized the Statistical Parametric Mapping 12 (SPM12; https://www.fil.ion.ucl.ac.uk/spm/software/spm12/) toolbox via MATLAB R2016b (Mathworks) for preprocessing. The Artifact Detection Toolbox (ART) in CONN 18b was used to identify sudden large head movements.

All retrieval scans were preprocessed using FSL 6.0.0 (FEAT; http://www.fmrib.ox.ac.uk/fsl). Pattern similarity between trials was computed using custom code programmed in RStudio version 1.2.5033 (RStudio Team, 2019; http://www.rstudio.com/).

All statistical testing was performed using RStudio version 1.2.5033 (RStudio Team, 2019; http://www.rstudio.com/), using custom code which can be found here: https://github.com/saudrain/paper-SCP2021

The following R packages were used for statistical analyses:
- stats (version 4.2.1)
- lme4 (version 1.1.30)
- lmerTest (version 3.1.3)
- car (version 3.1.0)
- nlme (version 3.1.158)
- psych (version 2.2.5)
- emmeans (version 1.7.5)
- Rmisc (version 1.5.1)

For manuscripts utilizing custom algorithms or software that are central to the research but not yet described in published literature, software must be made available to editors and reviewers. We strongly encourage code deposition in a community repository (e.g. GitHub). See the Nature Portfolio guidelines for submitting code & software for further information.

Data

Policy information about availability of data

All manuscripts must include a data availability statement. This statement should provide the following information, where applicable:

- Accession codes, unique identifiers, or web links for publicly available datasets
- A description of any restrictions on data availability
- For clinical datasets or third party data, please ensure that the statement adheres to our policy

As participants did not consent for public release of their MRI scans, we present the de-identified scores on behavioural tasks, as well as processed connectivity and pattern similarity values for each ROI analyzed. These data can be found at: https://github.com/saudrain/paper-SCP2021.

Field-specific reporting

Please select the one below that is the best fit for your research. If you are not sure, read the appropriate sections before making your selection.

☒ Life sciences ☐ Behavioural & social sciences ☐ Ecological, evolutionary & environmental sciences

For a reference copy of the document with all sections, see nature.com/documents/nr-reporting-summary-flat.pdf

Life sciences study design

All studies must disclose on these points even when the disclosure is negative.

Sample size

This study was powered on Tompary and Davachi's (2017) finding of increased pattern similarity in the mPFC over time for arbitrary object-scene pairs that share the same/overlapping scene, versus were paired with different/non-overlapping scenes. Specifically, we powered the study based on the interaction the authors described between time and stimulus overlap on pattern similarity in the mPFC ($F(1,18) = 7.33$, $p = 0.01$), as the role of the mPFC in integrating memories was the primary aim of our study.

As Tompary and Davachi (2017) did not report effect sizes for this analysis, we calculated partial eta-squared using their F statistic and degrees of freedom using the following formula:

Partial eta-squared = (F * DF1) / ((F * DF1) + DF2)

We calculated that the partial eta-squared for the effect of interest was 0.29, which is a large effect size for the interaction between time and stimulus overlap. As there is no way to convert partial eta squared to Cohen's f required for G*Power analysis, and no way to calculate Cohen's f based on the information provided in the Tompary and Davachi (2017) paper, we chose to approximate an effect size for the power analysis. Given the issue of publication bias and true effect sizes tending to be smaller than the original published findings, we used a smaller, medium effect size approximate for a cohen's f, of 0.25.

In G*power, we used the cohen's f (0.25) to estimate a repeated measures ANOVA within factors, with 1 group, and 4 measurements (delay(2) x congruency(2)), with 0.5 correlation among repeated measures. This analysis indicated that 23.13 subjects would achieve 80% power.

Data exclusions

Total percent correct was below chance (<33%) in four participants at the long delay, and, therefore, their data for the long delay was excluded in all analyses; data at the short delay were retained. For resting state connectivity analyses an additional 2 participants were excluded as resting state scans were not collected due to technical issues.

Replication

The behavioural results are a replication of results obtained from a small sample of participants during behavioural piloting. The neuroimaging results have not been replicated in an independent sample, because we have not acquired an independent dataset with which to replicate the data.

Randomization

In this within-subject design, all participants underwent testing in all experimental conditions. Participants were randomly allocated to one of two counterbalanced conditions to control for practice effects with repeated testing: Group A underwent memory testing across the short delay followed by memory testing across the long delay. Group B underwent testing across the long delay followed by testing across the short delay.

Blinding

The data analyst was not blinded to counterbalancing of delays as the counterbalancing was not an effect of interest in the study. Rather, data were averaged across counterbalancing conditions, to mitigate any effect of delay order on memory. Participants were not blinded because it would be impossible to blind participants to the counterbalanced group that they were in.

Reporting for specific materials, systems and methods

We require information from authors about some types of materials, experimental systems and methods used in many studies. Here, indicate whether each material, system or method listed is relevant to your study. If you are not sure if a list item applies to your research, read the appropriate section before selecting a response.

Materials & experimental systems

n/a	Involved in the study
☒	☐ Antibodies
☒	☐ Eukaryotic cell lines
☒	☐ Palaeontology and archaeology
☒	☐ Animals and other organisms
☐	☒ Human research participants
☒	☐ Clinical data
☒	☐ Dual use research of concern

Methods

n/a	Involved in the study
☒	☐ ChIP-seq
☒	☐ Flow cytometry
☐	☒ MRI-based neuroimaging

Human research participants

Policy information about studies involving human research participants

Population characteristics: Twenty-three young adults (8 Male /15 Female, mean age: 26.39 years, range: 22 – 34 years) participated in this experiment. All participants were English speakers with normal or corrected to normal vision, and no active diagnosis of neurological or psychiatric disorder.

Recruitment: Participants were recruited through the Human Subjects Participant Pool at the psychology department at the University of Toronto, through flyers at the University of Toronto campus, and through word of mouth. As such, most of the participants in this study are neurologically healthy young adults with high IQ. The extent to which these findings are generalizable to a broader population is therefore unclear. Any bias in selection is unlikely to effect the results across conditions in this experiment, as this is a within-participant design. All measures were taken with each individual participant.

Ethics oversight: The experimental protocol was approved by the University of Toronto Research Ethics Board.

Note that full information on the approval of the study protocol must also be provided in the manuscript.

Magnetic resonance imaging

Experimental design

Design type: event-related fMRI task design; resting-state fMRI

Design specifications: For each encoding session, participants were presented with images of 80 object-scene pairs one at a time, half of which were congruent. For each encoding session, they studied each object-scene pair three times across three 6.5 minute long encoding runs. All pairs were presented in each encoding run in a pseudo-random order for each participant, such that adjacent trials did not share the same scene. Participants viewed each scene for 0.1 seconds on its own before it was overlaid with the paired object for an additional 2 seconds. This brief temporal overlaying strategy was implemented to emphasize that the object and scene were separate entities rather than a unitized construct. They were then presented with a screen with response options for 1 second during which they indicated if the object-scene pair had been related or unrelated using an MRI compatible button box. The response window was followed by a jittered fixation period lasting 1, 1.5, or 2 seconds.

After each delay (10 minutes, 72 hours), participants underwent a cued retrieval session during which they viewed studied objects individually in the absence of the paired background scene, and were asked to retrieve the scene that had been paired with the object as vividly as possible. The 80 learned pairs were tested across four 4-minute runs with 20 objects presented in each run. Each object was presented for 2 seconds during which time participants were to visualize the paired scene. Participants were then shown a response screen and had 2 seconds to indicate with which context the object had been paired with (kitchen/beach/don't know). The response screen remained on for the duration of the 2 seconds regardless of the speed of the button press. If they indicated that the object had been paired with a kitchen or a beach, they were then shown another response screen for an additional full 2 seconds, during which they indicated with which specific beach or kitchen scene the object had been paired with (for example, if they chose "beach" they were offered the following response options: big beach/small beach/don't know). Objects were presented in a random order for each participant, and all responses were recorded using an MRI-compatible button box. Each trial ended with a jittered fixation period lasting 3 to 6 seconds.

Each participant underwent 2 resting-state scans. The first occurred at the very beginning of the first session, and the second occurred after encoding for the long delay. Rest scans were 6 minutes long, wherein participants were instructed to fixate on a small black cross in the center of a gray screen and remain awake.

Behavioral performance measures: Button presses recorded all behavioural responses. Participants were included in the analysis if the total percent correct score across conditions was above chance (>33%) for a given delay.

Eye-tracking during scanning was used to ensure participants remained awake during resting-state scans.

Acquisition

Imaging type(s) | functional; structural

Field strength | 3T

Sequence & imaging parameters | Functional echo-planar imaging (EPI) scans were oriented horizontally to intersect the anterior and posterior commissures (TR = 1.5s TR, TE = 26ms, flip angle = 70 degrees, FOV = 220x220, 52 slices, 2.5mm x 2.5mm x 3mm voxels), and were acquired with a GRAPPA acceleration factor of 1, and a multiband factor of 2. Phase encoding was in the anterior to posterior direction, with interleaved acquisition in the inferior to superior direction along the z-axis. A fieldmap scan was also collected, using a double-echo gradient echo sequence with the same parameters as the EPI sequence (with the exception of the following: TR = 0.88, TE1 = 4.92ms, TE2 = 7.38ms, flip angle = 60 degrees). A T1-weighted magnetization-prepared rapid-acquisition gradient echo (MPRAGE) sequence (1mm isotropic voxels, 160 sagittal slices) was also collected.

Area of acquisition | whole brain

Diffusion MRI ☐ Used ☒ Not used

Preprocessing

Preprocessing software | All retrieval scans were preprocessed using FSL 6.0.0 (FEAT; http://www.fmrib.ox.ac.uk/fsl).

Resting-state scans were preprocessed using CONN version 18b (https://web.conn-toolbox.org/).

Normalization | The hippocampal regions of interest were manually segmented for each participant's unique anatomy to improve accuracy and specificity, and as such, all analyses were conducted in native space.

Normalization template | The data were not normalized

Noise and artifact removal | The task-based fMRI data was de-noised during preprocessing using FSL FEAT. Head movement was estimated for each functional run (6 rigid body motion estimates corresponding to translations and rotations around x, y, and z-axes, which were saved as regressors for later modelling) and the EPI was realigned to correct for motion using FSL's MCFLIRT. Volumes with framewise displacement > 0.9 were flagged using FSL's motion outlier's tool, to be used as regressors during first level modelling in order to account for large changes in signal intensity that occur with sudden large head movements. To reduce spatial distortion of the EPI images, an unwrapped phase map in rad/s was constructed from the magnitude (skull-stripped) and phase fieldmap images, and applied to the EPI data simultaneously with motion correction to minimize interpolation-related image blurring.

For the resting-state scans, motion was estimated and realignment, unwarping, and distortion correction were applied to the EPI images simultaneously. Volumes contaminated by sudden large head movements were identified using the Artifact Detection Toolbox97 (ART), which flagged TRs with fluctuations in global signal greater than 3 standard deviations, translational motion greater than 1mm, and rotational motion greater than 0.05 radians. The EPI images were co-registered to the T1-weighted anatomical scan, and were segmented into grey matter, white matter, and cerebrospinal fluid masks for each participant. We used aCompCor to exclude physiological noise by regressing out the top five principal components from the data – as identified from a principal components analysis on the unsmoothed signal from eroded white matter and cerebral spinal fluid masks. The motion parameters (6 rigid body realignment parameters and their first order temporal derivatives, plus the high motion volumes identified by ART) were also regressed out, and the data were temporally filtered to exclude very low (<0.008 Hz) and high (>0.09 Hz) frequency fluctuations.

Volume censoring | For task fMRI data, volumes with framewise displacement > 0.9 were flagged using FSL's motion outlier software, and were used as regressors during first level modeling in order to account for large changes in signal intensity that occur with sudden large head movements.

For resting-state scans, volumes contaminated by sudden large head movements were identified using the Artifact Detection Toolbox (ART), which flagged TRs with fluctuations in global signal greater than 3 standard deviations, translational motion greater than 1mm, and rotational motion greater than 0.05 radians. These TR's were used as regressors of no interest during first level modeling.

Statistical modeling & inference

Model type and settings | We used representational similarity analysis for our task-fMRI data. All preprocessed retrieval scans were modeled in each participant’s native space. We took a Least Squares Single (LSS) pattern estimation approach, wherein each trial’s activation was estimated with a separate GLM. The first regressor in each model represented the trial of interest (specifically, the portion of the trial where the object was on the screen and the participant was remembering the paired associate), and five additional regressors modeled the remaining trials within the same run according to trial type (coarse congruent, coarse incongruent, detailed congruent, detailed incongruent, forgotten). There were additional regressors for each response window. Finally, in order to correct for head motion, there were 6 regressors for rigid body motion parameters (translations and rotations around x, y, and z-axes), as well as a regressor for each TR that was flagged as having greater framewise displacement than 0.9 during preprocessing. Regressors were convolved with a double gamma HRF. A map of t-values for the first parameter estimate was retained for each model and represents the activation for each trial during retrieval. For each trial, the spatial pattern of activity across each ROI was extracted into a vector and z-scored. Similarity between different

vectors was calculated with Pearson correlations, which were Fisher-transformed prior to statistical testing. To avoid inflated correlations due to temporal proximity within each run, correlations were limited to trials occurring in different runs. Trial-level similarity was estimated using linear mixed effects models with experimental conditions as fixed effects and a random intercept for each subject, using the 'lme4' package. Denominator degrees of freedom and p-values were estimated using the Satterthwaite approximation as implemented using the 'lmerTest' package in R (https://cran.r-project.org/web/packages/lmerTest/index.html), given that this method produces results with relatively low Type I error rates and gives the most comparable results to regular linear models.

For resting-state analyses, average timeseries across the unsmoothed voxels within each native-space ROI were used to compute a Pearson's correlation between the ROIs of interest for each participant (mPFC- anterior hippocampus). Correlation values were Fisher transformed, and the resulting values from the pre-encoding scan were subtracted from the post-encoding values for each participant. These post-pre difference scores in pairwise connectivity for each participant were then correlated with participants' percent correct retrieval scores (congruent/incongruent coarse/detailed retrieval), using a one-tailed test for our a-priori hypothesis, and two-tailed tests for exploratory correlations.

Effect(s) tested

We correlated post-encoding change in resting-state connectivity relative to baseline connectivity with behavioural retrieval scores across the long delay.

We ran a linear mixed effects model predicting within-context pattern similarity in the mPFC as a function of congruency and delay, with random intercepts for each subject, and weighted to model unequal variance across fixed effects. We also computed a linear mixed model predicting pattern similarity in the mPFC as a function of context (same context/across context) and delay (short/long), separately for congruent and incongruent pairs. Random intercepts were included for each subject, and weights were included to model unequal variance across fixed effects.

We submitted pattern similarity correlations extracted from the anterior hippocampus to a scene (same/similar/other scene) x congruency (congruent/incongruent) x delay (short/long) linear mixed model, with a random intercept for each subject.

We submitted pattern similarity correlations extracted from the posterior hippocampus to a scene (same/similar/other scene) x congruency (congruent/incongruent) x delay (short/long) linear mixed model, with a random intercept for each subject.

Specify type of analysis: ☐ Whole brain ☒ ROI-based ☐ Both

Anatomical location(s)

The right hippocampus was anatomically defined for each participant using FSL's automatic subcortical segmentation protocol (FIRST). Each participant's right hippocampus was manually segmented in native space along its long axis at the uncal notch to create anterior and posterior hippocampal ROIs.

The mPFC mask was constructed from combining areas A14m and A10m from the probabilistic Brainnetome atlas bilaterally in MNI space (https://atlas.brainnetome.org/). These ROIs are together relatively inclusive of the mPFC. We did not include some of the most ventral mPFC ROIs of the Brainnetome atlas due to a high degree of signal dropout in these areas in some of our participants, resulting in noisy signal. The resulting mPFC mask was warped into each participant's native space using FSL's FLIRT function.

Statistic type for inference
(See Eklund et al. 2016)

Average timeseries across the unsmoothed voxels within each native-space ROI were used to compute a Pearson's correlation between the ROIs of interest for each participant (mPFC- anterior hippocampus) in the resting-state connectivity analysis. Associations between connectivity and behavioral measures were performed using Pearson's correlations, as described above.

For each trial of the task fMRI analysis, the spatial pattern of activity across each ROI was extracted into a vector and z-scored to be used for subsequent RSA analyses. Differences in pattern similarity were tested using linear mixed effects models as described above.

Correction

The results reported are uncorrected apriori hypotheses.

Models & analysis

n/a	Involved in the study
☐	☒ Functional and/or effective connectivity
☒	☐ Graph analysis
☐	☒ Multivariate modeling or predictive analysis

Functional and/or effective connectivity

Pearson's correlation

Multivariate modeling and predictive analysis

Dependent variables: pattern similarity in a given ROI
Independent variables: congruency (congruent/incongruent), delay (short/long), scene (same/similar/other), context (within/across)
random effects: participants
